# Poster Session I - A28 MUCUS FROM A HYPERSECRETORY MUTANT GOBLET-LIKE CELL INCREASES *ENTAMOEBA HISTOLYTICA* VIRULENCE AND CYTOTOXICITY

**DOI:** 10.1093/jcag/gwaf042.028

**Published:** 2026-02-13

**Authors:** H Gorman, F Moreau, M C Ruyechan, K Chadee

**Affiliations:** University of Calgary, Calgary, AB, Canada; University of Calgary, Calgary, AB, Canada; University of Calgary, Calgary, AB, Canada; University of Calgary, Calgary, AB, Canada

## Abstract

**Background:**

MUC2 mucin is produced by colonic goblet cells and forms a mucus bilayer that provides a physical barrier between potential pathogens in the lumen and the underlying epithelial cells. MUC2 is heavily glycosylated with a variety of glycans that imparts its functional properties. Mucus is the first line of innate host defense in the gut that colonic pathogens such as the parasite *Entamoeba histolytica* (*Eh*) degrades to cause amoebic colitis. As modifications in MUC2 mucus structure/function is a predisposing factor in *Eh* disease pathogenesis, we investigated how alterations in MUC2 glycans affected epithelial barrier functions against *Eh*.

**Aims:**

The specific aims are:

1. To quantify hypersecretory mucus barrier protection in response to *Eh*

2. To quantify *Eh* virulence factor expression in response to hypersecretory MUC2 mucin

**Methods:**

Two goblet cells lines were used; WT LS174T cells and a hypersecretory *Mut* LS174T clone selected using an unbiased screen. Whole genome sequencing revealed that *Mut* cells had mutations that upregulated the expression of *TP53*, *AGR2* and *MEK/MAPK* that enhanced MUC2 production with an altered glycomics profile. MUC2 secretion in WT and *Mut* cells was quantified by ^3^H-metabolically labeled mucus secretion in response to *Eh*. Integrity of the mucus layer was enumerated by *Eh* adherence to and cytotoxicity of cells measured by LDH release. *Eh* degradation of mucus was quantified using mucin specific antibodies against MUC2 protein and glycans by immunoblotting. *Eh* mucophagy of WT or *Mut* mucus was quantified by confocal microscopy and imaging flow cytometry. Following mucophagy, modulation of *Eh* virulence genes was measured by RT-PCR and enzymatic activity quantified using enzyme specific substrates.

**Results:**

*Mut* cells constitutively produced and secreted significantly more MUC2 mucin in response to *Eh* as compared to WT cells. Even though *Mut* mucus was more adherent to *Eh* than WT mucus it was not protective resulting in increased cell cytotoxicity. *Mut* mucus was dispersed and easily disrupted and ingested (mucophagy) by *Eh* as compared to dense packed polymeric WT mucus. The higher ingestion of *Mut* mucus by *Eh* significantly upregulated parasite virulence genes with increased enzymatic activity of secreted proteinases that degraded *Mut* mucus in a time- and dose-dependent manner.

**Conclusions:**

This study highlights the importance of mucus glycans in conferring structural and functional integrity of the mucus gel that provide barrier protective properties to the colonic epithelium. Understanding both quality and quantity of mucus may be an important predisposing factor against pathogen invasion and in GI disorders.

**Funding Agencies:**

CIHR

